# Plasma microRNAs as Biomarkers for Predicting Radiotherapy Treatment-Induced Cardiotoxicity in Lung Cancer

**DOI:** 10.3390/life14121619

**Published:** 2024-12-06

**Authors:** Paulina Kazlauskaitė, Ieva Vaicekauskaitė, Jonas Venius, Rasa Sabaliauskaitė, Rita Steponavičienė

**Affiliations:** 1 National Cancer Institute, 08406 Vilnius, Lithuania; 2 Institute of Biomedical Sciences, Faculty of Medicine, Vilnius University, 08661 Vilnius, Lithuania; 3 Institute of Biosciences, Life Sciences Center, Vilnius University, 10257 Vilnius, Lithuania

**Keywords:** non-small-cell lung cancer, RIHD, microRNA

## Abstract

**Background:** Lung cancer is the second most common malignancy and stands as a leading cause of cancer-related deaths worldwide. Currently, one of the main treatment options for lung cancer is radiotherapy, but this treatment is associated with complications, such as an increased risk of cardiac-related morbidity and mortality. However, currently available methods for predicting radiation-induced heart disease (RIHD) remain suboptimal. **Methods:** In this pilot study, using the RT-qPCR method, we analyzed the expression levels of six miRNAs (miRNA-1-3p, miRNA-21-5p, miRNA-24-3p, miRNA-29a-3p, miRNA-34a-5p, and miRNA-222-3p). **Results:** Fourteen pairs of locally advanced non-small-cell lung cancer patients’ plasma samples, taken before and after radiotherapy, were examined. It was observed that miRNA-1-3p, miRNA-21-5p, miRNA-24-3p, miRNA-29a-3p, and miRNA-222-3p were downregulated, while miRNA-34a-5p was upregulated in lung cancer patients’ plasma after treatment. Additionally, after definitive radiotherapy, patients with an increased NT-proBNP value displayed a statistically significant difference in miRNA-222-3p levels compared to the normal range of this indicator. The panel of the combined four miRNAs for assessing the risk of cardiac comorbidities demonstrated an AUC of 0.79, sensitivity of 71.43%, and specificity of 100%, with further improved values upon integration with clinical biomarker NT-proBNP. **Conclusions:** This pilot study shows that the identification of changes in miRNA expression levels in lung cancer patients’ plasma before and after radiotherapy could be used for the early diagnosis of RIHD.

## 1. Introduction

Lung cancer is the second most common malignancy and is the leading cause of cancer-related deaths worldwide [1]. In 2017, nearly a quarter of a million people died from lung cancer in Europe, which was one-fifth of all deaths from cancer and 5.1% of the total number of deaths [2].

Chemotherapy, either concurrent or sequential with radical radiotherapy, is the main treatment modality for early-stage and locally advanced non-operable lung cancer. However, its application has been associated with an increase in mortality rates due to complications arising from the treatment [3]. Systemic treatment caries within itself cardiovascular disorders. For example, cisplatin has been linked to an increased incidence of pulmonary arterial hypertension [4].

Recent studies have associated thoracic non-small-cell lung cancer (NSCLC) radiation therapy to an increased risk of cardiovascular-related morbidity and mortality, examining heart toxicity as a late side effect of multimodality treatment. In a recent study, the initiation of atrial fibrillation was associated with the radiation dose to the pulmonary veins during treatment of NSCLC [5]. In another research, an increasing heart dose was independently associated with an increased cardiac event rate and worse overall survival, serving as a dosimetric predictor in locally advanced NSCLC patients undergoing definitive radiotherapy [6]. A similar study demonstrated that the cumulative incidence of grade ≥ 3 cardiac events exceeded 10% within 24 months among patients with locally advanced NSCLC treated with definitive radiotherapy [7].

The guidelines from the American Heart Association (AHA) and the Heart Failure Society of America (HFSA) propose two biomarkers, troponin (Tn I) and natriuretic peptide (N-terminal pro B-type natriuretic peptide, NT-proBNP), for assessing heart failure [8]. Nevertheless, these biomarkers have their own drawbacks. Troponins face challenges in diagnosing acute myocardial infarctions due to their elevation in other conditions, such as direct chest trauma, that can be associated with the significant release of Tn I [9]. Furthermore, chronic kidney disease (CKD) [10] and increasing age [11] complicate the measurement of troponins in diagnosing acute myocardial infarctions. Similarly, the NT-proBNP test exhibits low specificity (not exceeding 0.50) and is not recommended for identifying heart disease in patients with either a history of cancer or active cancer [12]. Conditions like systemic inflammation [13], weight loss [14] and deterioration in renal function [15] are associated with an increased NT-proBNP, complicating its interpretation. The limitations of these biomarkers highlight the need for the identification and development of new, more suitable biomarkers to enhance the early prognosis of RIHD.

Recently, various potential biomarkers used for assessing RIDH are intensively studied. One such biomarker is microRNAs (miRNAs)—small non-coding single-stranded RNA molecules approximately 21–23 nt in length [16]. A key feature of these small RNAs is the control of gene expression. MiRNAs play a crucial role in regulating essential cellular functions, including development, differentiation, growth, and metabolism [17]. MiRNA expression changes in extracellular body fluids, including blood plasma [18], can indicate the possible presence of cancer or cardiovascular disease and are remarkably stable [19,20]. Recent studies have linked specific miRNAs with heart pathologies, such as myocardial infarction, arrhythmias (miRNA-1-3p, miRNA-34a-5p) [21,22], fibrosis (miRNA-21-5p, miRNA-29a-3p, miRNA-222-3p) [23,24,25,26], ischemia, and coronary heart disease (miRNA-24-3p) [27,28]. For instance, increased miRNA-21-5p levels have been associated with myocardial hypertrophy and cardiac fibrosis, as this miRNA is abundantly expressed in fibroblasts [23,29]. Similarly, decreased levels of miRNA-29a-3p with tumor progression have also been associated with cardiac fibrosis [30,31]. Furthermore, previous studies have reported that anticancer treatments, including radiotherapy, modulate the expression of the aforementioned miRNAs, and these alterations might be involved in cardiac function impairments (Appendix A, Table A1).

Considering these properties, miRNAs emerge as a promising candidate for developing new cancer tests for personalized treatment selection, disease diagnosis, and predicting response to therapy [19,32]. The evaluation of changes in miRNA levels could improve the prognosis of RIHD, risk stratification, and precise treatment planning, thereby reducing the observed cardiotoxicity in lung cancer patients receiving radiotherapy.

Although there are a number of publications studying changes in miRNA expression after radiotherapy, there is a lack of publications describing miRNA’s role in non-small-cell lung cancer during radiotherapy and monitoring treatment-induced cardiotoxicity. Therefore, the aim of this study was to identify changes in miRNA expression and their association with clinical features in the plasma, taken from lung cancer patients, pre- and post-ionizing radiation treatment, to evaluate the effects of treatment on the heart and identify miRNAs, which might be used to predict response to treatment.

## 2. Materials and Methods

### 2.1. Research Objective

Fourteen pairs of non-small-cell lung cancer patients’ plasma samples, collected before and after radiotherapy, were included in this study. All patients received a total radiation dose of 60 Gy to the tumor and metastatic lymph nodes. Blood plasma samples were collected at the Lithuanian National Cancer Institute between 2021 and 2022. All patients signed a patient consent form, which informed the subjects about the study and data collection. The research protocol was approved by the Lithuanian Bioethics Committee (No. 158200-18/7-1034-540 amendment No. 1).

From the 14 studied patients, 21% (3/14) were female, and the average age was 72 with a standard deviation of 5 years. A total of 60% (6/10) had an increased NT-proBNP value, and only 10% (1/10) had an increased Tn I value after radiotherapy. Additionally, 69% (9/13) of patients had cardiac comorbidities, and 57% (8/14) used at least one type of cardiovascular medication regularly. Lastly, 57% (8/14) of all patients had a malignant neoplasm of the upper lobe, bronchus, or lung; 29% (4/14) had an overlapping malignant neoplasm of the bronchus and lung; and only 14% (2/14) had a malignant neoplasm of the middle lobe, bronchus, or lung. The clinical features of the patients included in the study are provided in Table 1.

### 2.2. Sample Collection

Blood was collected from each patient before and after radiotherapy treatment. The blood samples were collected from the patients’ veins and centrifuged (Heraeus Megafuge 8R, Thermo Fisher Scientific, Osterode am Harz, Germany) at 1000× *g* for 15 min. The blood plasma was collected in 1.5 mL cryotubes, and the samples were stored at −80 °C until miRNA extraction.

### 2.3. miRNA Extraction

Total RNA, including miRNA, from the plasma samples before and after treatment with ionizing radiation, was isolated with miRNeasy Serum/Plasma Kit (QIAGEN, Hilden, Germany) according to the manufacturer’s protocol (January 2020). During RNA extraction, 3.5 μL of 0.5 nM diluted miRNeasy Serum/Plasma Spike-In Control (*C. elegans* miRNA-39-3p miRNA mimic) was added. The quantity and quality of the extracted RNA were evaluated using a Nanodrop 2000 spectrophotometer (Thermo Fisher Scientific, Wilmington, DE, USA). Samples were then stored at a temperature of −80 °C until use.

### 2.4. cDNA Synthesis

Complementary DNA (cDNA) was synthesized from the RNA samples following the manufacturer’s recommended protocol using the TaqMan^®^ Advanced miRNA cDNA Synthesis Kit (Thermo Fisher Scientific, Carlsbad, CA, USA) on the ProFlex™ 3 × 32–well PCR System (Applied Biosystems, Singapore). In brief, 2 μL of total RNA extracted from the patient’s plasma was first polyadenylated using poly(A) polymerase from the kit, and the 5’ end of the RNA was lengthened through an adapter ligation step. The modified RNA was then reverse-transcribed into cDNA using universal reverse transcription primers. Finally, the resulting cDNA was uniformly amplified for miRNA enrichment. The synthesized cDNA was stored at −80 °C or further used for RT-qPCR.

### 2.5. Quantitative PCR

The levels of six miRNAs (miRNA-1-3p, miRNA-21-5p, miRNA-24-3p, miRNA-29a-3p, miRNA-34a-5p, and miRNA-222-3p) were analyzed. An exogenous control, cel-miRNA-39-3p, was included in the samples during miRNA extraction. Quantitative PCR (qPCR) was prepared using TaqMan^®^ Universal PCR Master Mix [2X] (Thermo Fisher Scientific, Vilnius, Lithuania), specific miRNA primers (TaqMan^®^ Advanced MicroRNA Assays, (Thermo Fisher Scientific, Pleasanton, CA, USA), and nuclease-free water according to the manufacturer’s protocol. Subsequently, diluted synthesized cDNA template (dilution ratio 1:10) was added to the PCR reaction plate. The reverse transcription quantitative polymerase chain reaction (RT-qPCR) was conducted using a QuantStudio™ 5 Real-Time PCR Instrument (Thermo Fisher Scientific, Singapore) according to the standard amplification protocol provided with Universal PCR Master Mix.

### 2.6. Statistical Analysis

Cycle threshold (Ct) values from RT-qPCR data were normalized with an exogenous cel-miRNA-39-3p control, and relative expression was acquired by calculating comparative Ct ($\log_{2}\left(2^{-\Delta \Delta \mathrm{Ct}}\right)$) [33]. These values reflected the difference in miRNA levels before and after radiotherapy in the blood plasma of lung cancer patients and were used for further statistical analysis. Statistical analysis and visualization were performed using computer programs R version 4.3.2 (R Core Team, Vienna, Austria, 2023) and RStudio version 2023.09.1+494 (Posit team, Boston, MA, USA, 2023). Data normality was assessed using the Shapiro-Wilk test. If the data met the condition of normality, further analysis was conducted using Welch’s *t*-test [34,35]. The ROC (Receiver Operating Characteristic) analysis was used to evaluate biomarker sensitivity and specificity. Logistic-regression-predicted probabilities were used to combine the panel of multiple miRNAs. The DeLong test was used to compare the areas under the curve (AUC) of ROC curves [36]. Results were considered statistically significant if *p* < 0.05.

## 3. Results

### 3.1. Concentration and Purity of miRNA

Total RNA was extracted from 200 μL blood plasma samples, with concentrations ranging from 10.90 to 49.50 ng/μL (median 19.20 ng/μL, interquartile range 12.23 ng/μL). The average A260/280 absorbance ratio was 1.42 (standard deviation 0.06), while the median of A260/230 absorbance ratio was 0.46 (interquartile range 0.30).

### 3.2. miRNA Expression Changes After Treatment

**Figure 1:**
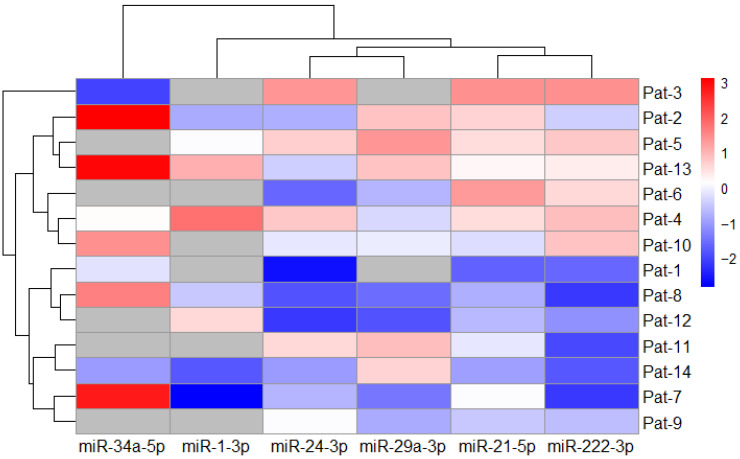
Heatmap of miRNA expression fold change after treatment (*n* = 14). Gray cells indicate no data.

The fold change analysis showed that miRNA-1-3p, miRNA-21-5p, miRNA-24-3p, miRNA-29a-3p, and miRNA-222-3p were downregulated, while miRNA-34a-5p was upregulated in lung cancer patients’ plasma after radiotherapy (Figure 1). Such results indicate that the levels of selected miRNAs differed after treatment period, with the highest relative expression of miRNA-34a-5p and the lowest relative expression of miRNA-24-3p, respectively.

### 3.3. Changes in miRNA Expression in Relation to Disease Diagnosis

**Figure 2:**
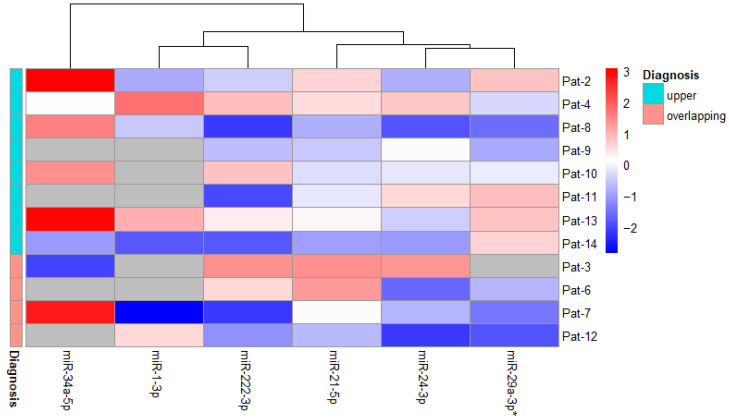
Heatmap of miRNA expression fold change after treatment among patients diagnosed with malignant neoplasm of the upper lobe, bronchus, or lung (*n* = 8) and those diagnosed with overlapping malignant neoplasm of the bronchus and lung (*n* = 4). The group diagnosed with malignant neoplasm of the middle lobe, bronchus, or lung is not shown due to its small sample size. Gray cells indicate no data. “*”—indicates a statistically significant value.

This study also examined the impact of lung cancer diagnosis on miRNA levels. Patients were categorized based on three primary diagnoses: malignant neoplasm of the upper lobe, bronchus, or lung; malignant neoplasm of the middle lobe, bronchus, or lung, and overlapping malignant neoplasm of the bronchus and lung, affecting multiple specific sites within the body. After categorizing the alterations in miRNA levels based on the most prevalent diagnoses, it was observed that the quantity of miRNA-21-5p varied depending on the tumor diagnosis, transitioning from downregulation to upregulation. However, these detected changes were not significant (Figure 2).

Divergent outcomes were observed in miRNA-29a-3p levels. In malignant tumors of the upper lobe, bronchus, or lung, miRNA-29-3p levels were higher compared to the cases where the tumor had already metastasized, resulting in a decrease in miRNA expression. The decrease in miRNA-29a-3p levels based on diagnosis was statistically significant (*p* = 0.026) (Figure 2).

### 3.4. Correlation of miRNA Expression and Regular Medication Use

**Figure 3:**
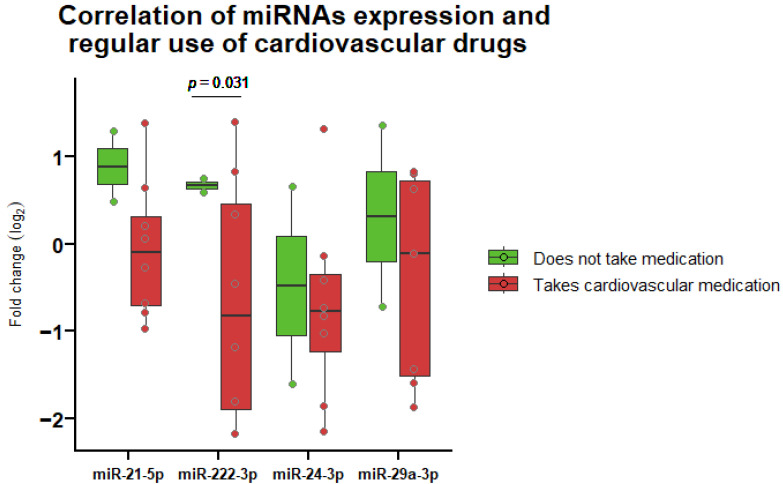
Correlation between miRNA expression fold change after therapy and regular (non-)use of cardiovascular drugs (*n* = 10). MiRNA-1-3p and miRNA-34a-3p are not shown due to missing data. Boxes indicate the $\log_{2}$ normalized expression data, and the line inside each box represents the median. Whiskers denote the minimum and maximum values.

All miRNA expression after radiotherapy was found to be reduced in patients taking at least one type of cardiovascular medication regularly. Our patients took FDA-approved drugs, such as atorvastatin, nebivolol, etc., prescribed for preventing heart attack by reducing total blood cholesterol [37] or treating hypertension [38]. A statistically significant value (*p* = 0.031) was found for miRNA-222-3p among patients (non-)users of regular medication (Figure 3). No significant changes in miRNA-222-3p levels or other studied miRNAs were found between the groups of patients who took at least one cardiovascular medication and those who took other medication not related to the treatment of cardiovascular disease, or those who took other medication or no medication at all.

### 3.5. Correlation of miRNA Expression and Clinical Biomarker of Heart Damage

**Figure 4:**
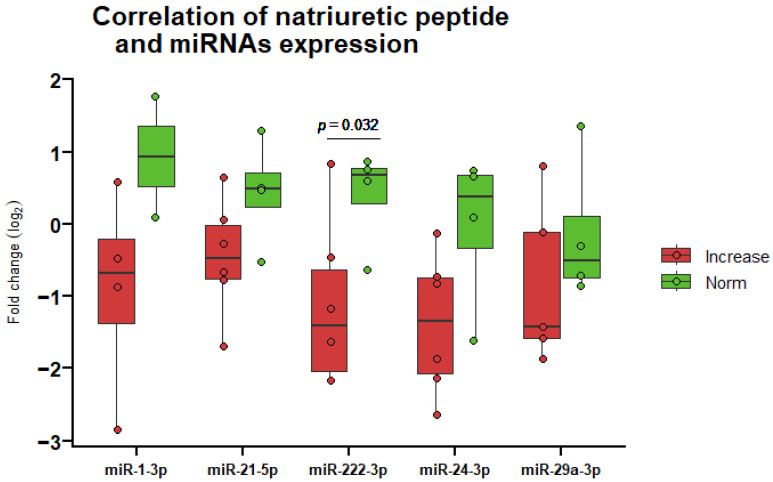
MiRNA expression fold change after radiotherapy in patients with an increase in natriuretic peptide concentration versus non-increase (norm) (*n* = 10). MiRNA-34a-3p is not shown due to missing data. Boxes indicate the $\log_{2}$ normalized expression data, and the line inside each box represents the median. Whiskers denote the minimum and maximum values.

The changes in miRNA levels were compared with the clinical biomarker N-terminal B-type natriuretic peptide (NT-proBNP), used to assess heart damage. It was observed that after radiotherapy, patients with an increased NT-proBNP value displayed statistically significant differences in miRNA-222-3p (*p* = 0.032) levels compared to the normal range of this indicator (Figure 4).

Due to missing data and a small sample size, we did not compare miRNA levels with the clinical biomarker troponin I (Tn I) since an increased value was observed in only one patient (Table 1).

### 3.6. Correlation of miRNA Expression and Cardiac Comorbidities

Finally, we evaluated the correlation between miRNA expression levels after radiotherapy and concomitant cardiovascular diseases in patients. The most common diagnoses among patients were treatment-related cardiovascular diseases, including hypertensive heart disease, myocardial infarction, ischemic heart disease and conduction disorders.

**Figure 5:**
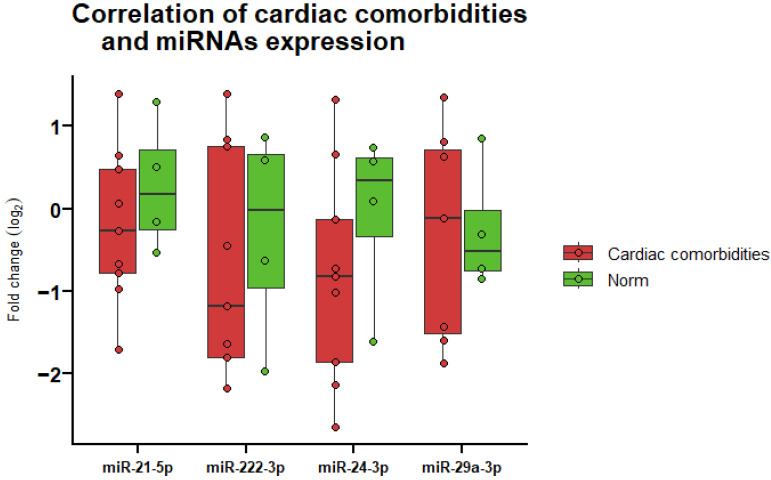
MiRNA expression fold change after radiotherapy in patients with cardiac comorbidities versus without (norm) (*n* = 13). MiRNA-1-3p and miRNA-34a-5p were excluded from the analysis due to missing data. Boxes indicate the $\log_{2}$ normalized expression data, and the line inside each box represents the median. Whiskers denote the minimum and maximum values.

After treatment, the expression of all miRNAs was found to be reduced in patients with treatment-related cardiovascular diseases. Nevertheless, no statistically significant differences in miRNA expression were observed between the presence and absence of concomitant cardiovascular diseases (Figure 5).

**Figure 6:**
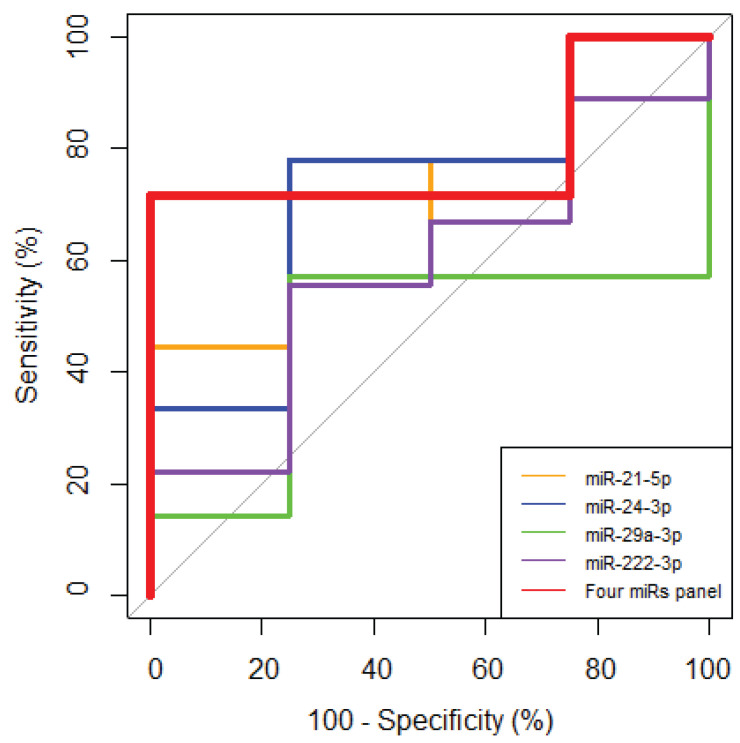
ROC curve analysis of the miRNA expression fold change in relation to cardiac comorbidities after radiotherapy (*n* = 13). MiRNA-1-3p and miRNA-34a-5p were excluded from the analysis due to missing data.

The ability of each investigated miRNA to distinguish clinically significant samples after treatment from those at risk of concomitant cardiovascular diseases was assessed using Receiver Operating Characteristic (ROC) curves. Among the studied miRNAs, miRNA-24-3p was the best predictor of cardiac comorbidities, which successfully separated treatment-related cardiovascular diseases after radiotherapy with an AUC of 0.69, sensitivity of 77.78%, and specificity of 75% (Figure 6 and Table 2). Additionally, a combined panel, comprising four miRNAs (miRNA-21-5p, miRNA-24-3p, miRNA-29a-3p, and miRNA-222-3p), was developed. The panel of combined four miRNAs for assessing the risk of cardiac comorbidities demonstrated an AUC of 0.79, sensitivity of 71.43%, and specificity of 100%. A DeLong test comparing the ROC curves for miRNA-24-3p and the four-miRNA panel showed a *p* value of 0.832.

**Figure 7:**
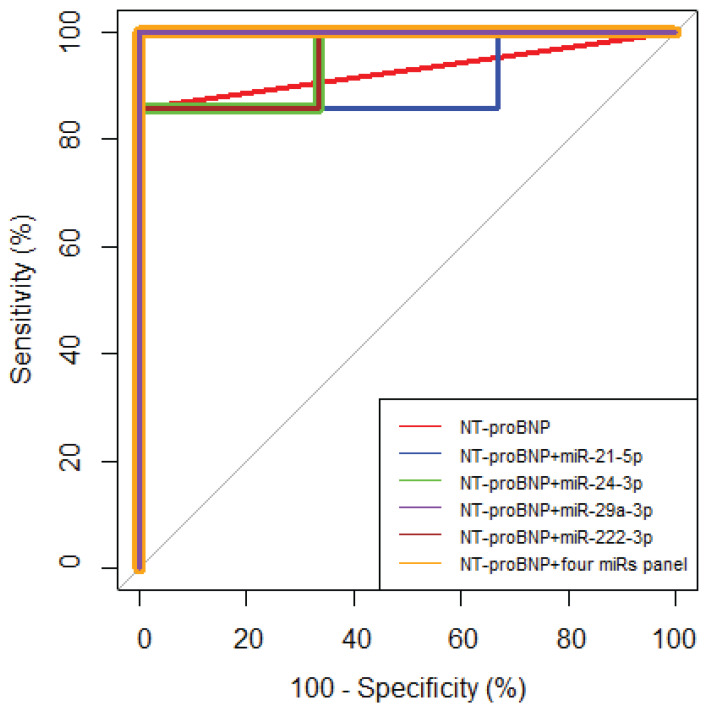
ROC curve analysis of miRNA expression fold change and NT-proBNP status in relation to cardiac comorbidities after radiotherapy (*n* = 10). MiRNA-1-3p and miRNA-34a-5p were excluded from the analysis due to missing data.

In addition, we evaluated the diagnostic efficacy of each miRNA by comparing their values with the clinical biomarker NT-proBNP status. Subsequently, we explored the combined diagnostic potential of miRNAs and NT-proBNP. The combined four-miRNA (miRNA-21-5p, miRNA-24-3p, miRNA-29a-3p, and miRNA-222-3p) panel along with NT-proBNP, or the combination of only miRNA-29a-3p with NT-proBNP, demonstrated an AUC of 1, sensitivity of 100%, and specificity of 100% (Figure 7 and Table 3). This signifies a 100% probability of distinguishing the class with comorbid cardiovascular disease risk from the class without. This comprehensive analysis revealed that integrating NT-proBNP with miRNAs enhanced diagnostic predictions associated with cardiac comorbidities following radiotherapy treatment. Interestingly, the investigated miRNAs augmented the clinical biomarker NT-proBNP’s efficacy and improved its capacity to differentiate between groups with comorbid cardiovascular disease risk more effectively than NT-proBNP alone. However, a DeLong test comparing the ROC curves for NT-proBNP and its combination with miRNAs showed no statistically significant differences (*p* > 0.05).

## 4. Discussion

Throughout this study, we assessed alterations in the levels of miRNA-1-3p, miRNA-21-5p, miRNA-24-3p, miRNA-29a-3p, miRNA-34a-5p, and miRNA-222-3p in the blood plasma of non-small-cell lung cancer patients before and after radiotherapy. These changes were then compared with the clinical data of the patients to evaluate the treatment’s impact on cardiotoxicity.

We successfully isolated total RNA, including miRNA, using the commercial miRNeasy Serum/Plasma Kit (QIAGEN, Germany). Previous studies have highlighted key challenges in miRNA studies, including the low concentration of miRNA in blood, which requires specialized kits for optimal miRNA recovery. Additionally, since miRNAs were extracted from biofluids, their low concentration in the aqueous phase and the high levels of blood proteins complicate the isolation process [39]. These challenges are reflected in low A260/280 and A260/230 absorbance ratios, indicating contamination. Other studies using the same kit have reported similar total RNA concentrations and purity from all of the blood samples [40], further showing difficulties of achieving optimal miRNA extraction. Furthermore, due to the low amount of miRNAs isolated from the blood plasma, a pre-amplification step of cDNA was included in our study to enhance miRNA detection.

Our analysis of blood plasma samples from lung cancer patients undergoing radiotherapy revealed a decrease in the expression of miRNA-1-3p, miRNA-21-5p, miRNA-24-3p, miRNA-29a-3p, and miRNA-222-3p, along with an increase in miRNA-34a-5p expression. In comparison, in a study closest to our design, Dinh et al. examined plasma samples from five patients with NSCLC during radical thoracic radiotherapy and observed a decrease in miRNA-29a-3p with increasing radiation dose, which aligns with our results [41]. Furthermore, Hu et al. determined that miRNA-34a was significantly increased in human cardiomyocytes exposed to radiation, providing additional insights that corroborate our findings [42]. Moreover, Esplugas et al. studied blood samples from breast cancer patients who underwent radiotherapy, highlighting that among all examined miRNAs, miRNA-222 exhibited the highest expression, leading them to conclude that radiotherapy promoted miRNA-222 overexpression [43]. In other studies with model organisms, Kura et al. observed an irradiation-induced downregulation of miRNA-1 in the myocardium of rats exposed to a single dose of 25 Gy. However, they noted a divergent upregulation of miRNA-21 in irradiated hearts, non-treated with drugs [44]. Additionally, studies in rats demonstrated a significant increase in myocardial miRNA-21 in both the left and right ventricles of post-irradiated rats compared to the non-irradiated controls [29]. Despite the parallels, neither of these two studies investigated the presence of cancer. Finally, Kang and colleagues, who applied RT-qPCR analysis to assess miRNA-24 expression in mice bearing nasopharyngeal carcinoma tumors treated with radiation therapy, showed an increase in levels of miRNA-24 [45]. While our findings align with certain trends in previous studies, it is important to note that the expression patterns of miRNAs post-irradiation vary across different experimental designs and cohorts. Moreover, many of these studies did not sample patients with cancer, and often, this important aspect of the studies was not considered. This underscores the need for further research to elucidate the context-dependent nature of miRNAs’ response to radiotherapy in the presence of cancer. The summarized studies can be found in the Appendix A, Table A1.

Previous studies have shown that our investigated miRNAs are important in cancer-related processes, such as cell proliferation [46], migration [47], invasion [48], epithelial–mesenchymal transition [49], and apoptosis [50]. Moreover, studies have indicated that miRNA-24 can function as either a tumor suppressor or an onco-miRNA in different cancer types, or even in the same cancer type by targeting different targets [51]. Our findings revealed that the relative expression of two miRNAs, miRNA-21-5p and miRNA-29a-3p, varied based on the diagnosis. These results suggest a hypothesis that both miRNAs could play a double role in human cancers. They could act as tumor suppressors when downregulated or as onco-miRNAs when upregulated, particularly in the context of cancer metastasis. Our study shows that when assessing changes in miRNA levels after radiotherapy treatment, it is important to consider the diagnosis of lung cancer patients as a factor that affects miRNA expression.

When comparing the correlations between the changes in miRNA expression and patients’ regular medication use, we observed that the continuous use of at least one type of cardiovascular drug influenced miRNA levels. Across all studied miRNAs, the total miRNA fold change after radiotherapy decreased with regular medication use, with a statistically significant decrease in miRNA-222-3p. These results align with previous studies. It has also been demonstrated that drugs, like nebivolol, used to treat hypertension, affect miRNA expression [52]. Moreover, in studies involving irradiated rats’ myocardium, treatment with atorvastatin resulted in reduced miRNA-1 and increased miRNA-21 expression [44]. Similarly, when the endothelial cells of human umbilical veins were exposed to atorvastatin, a decrease in the expression of miRNA-222 was observed [53]. These findings further support the observed significant downregulation of miRNA-222 by cardiovascular drugs in our study.

By examining the correlation between miRNA levels and a clinically utilized biomarker that indicates heart damage (NT-proBNP), a statistically significant association was identified. This may indicate that the studied miRNAs can potentially assess the risk of heart damage in patients after radiation treatment, and their altered level in blood plasma after treatment may be related to various causes and complications of heart damage. Comparatively, Su et al. highlighted the diagnostic potential of circulating miRNA-1 in early acute myocardial infarction (AMI), showing almost equal diagnostic potential to Tn I, with improved accuracy when miRNA-1 was combined with Tn I [54]. Additionally, Zhang et al. demonstrated a correlation between circulating miRNA-21 and Tn I in patients with AMI [55]. Studies by Ntelios et al. focusing on hypertrophic cardiomyopathy patients revealed that miRNA-29a plasma levels did not correlate with serum Tn I levels [56]. Regrettably, our study encountered limitations due to missing data and its small sample size, precluding a direct comparison of the diagnostic potential of the studied miRNAs with that of Tn I. Furthermore, Sieweke et al. found that miRNA-21 and NT-proBNP correlated with echocardiographic parameters and could predict atrial fibrillation [57]. Lastly, after performing correlation analyses, Zhou and colleagues reported that NT-proBNP levels were positively correlated with the miRNA-222 level in patients with degenerative valvular heart disease [58]. In our investigation, we similarly observed that the studied miRNAs enhanced the diagnostic potential and accuracy of NT-proBNP, surpassing the performance achieved by NT-proBNP alone. Notably, previous studies focused solely on heart diseases and lacked the inclusion of cancer patients, revealing a critical gap in research that integrates both aspects.

Owing to the design of our pilot study, our analysis was constrained by the limited number of blood plasma samples. The relatively small sample cohort posed a challenge to the statistical power to test miRNA expression changes following radiotherapy and their correlation with clinical data. While our findings provide valuable preliminary insights, they should be critically evaluated because of these limitations. To enhance the reliability and generalizability of our findings, it is necessary to expand the sample size in future studies, allowing for a more comprehensive validation of our results.

## 5. Conclusions

The treatment of locally advanced inoperable non-small-cell lung cancer is multimodal, including chemotherapy, radiotherapy, and, for PDL-positive patients, immunotherapy. Early detection and treatment of possible cardiovascular toxicity is crucial for successful multimodal treatment continuation. Our pilot study suggests that the identification of miRNA-expression-level changes in lung cancer patients’ plasma before and after treatment could be developed into a new test for the early diagnosis of RIHD. However, further analysis is needed to validate these miRNAs as potential biomarkers for radiotherapy treatment-induced cardiotoxicity.

## Figures and Tables

**Table 1:** Clinical features of the patients included in the study.

Patient ID	Sex	Age	NT-proBNP	Tn I	Cardiac Comorbidity	Diagnosis	Regular Use of Cardiovascular Medication
Pat-1	Female	71	↑^1^	Norm	+ ^2^	Middle ^4^	Other ^7^
Pat-2	Female	81	↑	Norm	+	Upper ^5^	+
Pat-3	Male	68	No data	No data	+	Overlapping ^6^	+
Pat-4	Male	62	Norm	Norm	−^3^	Upper	Other
Pat-5	Male	68	Norm	Norm	+	Middle	−
Pat-6	Male	65	Norm	Norm	−	Overlapping	−
Pat-7	Male	77	↑	Norm	+	Overlapping	+
Pat-8	Male	72	↑	↑	+	Upper	+
Pat-9	Male	67	Norm	Norm	−	Upper	Other
Pat-10	Female	76	↑	Norm	+	Upper	+
Pat-11	Male	75	No data	No data	−	Upper	Other
Pat-12	Male	78	↑	Norm	+	Overlapping	+
Pat-13	Male	70	No data	No data	No data	Upper	+
Pat-14	Male	73	No data	No data	+	Upper	+

^1^ “↑”—indicates an increased value after treatment; ^2^ “+”—indicates the presence of the disease or regular medication use; ^3^ “−”—indicates the absence of the disease or no medication use; ^4^ “Middle”—malignant neoplasm of the middle lobe, bronchus, or lung; ^5^ “Upper”—malignant neoplasm of the upper lobe, bronchus, or lung; ^6^ “Overlapping”—overlapping malignant neoplasm of the bronchus and lung; ^7^ “Other”—indicates medications that are not prescribed to treat medical conditions associated with the heart; Norm Tn I was <0.017 μg/L; Norm NT-proBNP was <133 ng/L; NT-proBNP and Tn I values were evaluated after radiotherapy.

**Table 2:** ROC curve analysis characteristics of biomarkers in relation to cardiac comorbidities after radiotherapy (*n* = 13). AUC—area under the curve; CI—confidence interval; PPV—positive predictive value; NPV—negative predictive value.

Panel	AUC	95% CI	Sensitivity, %	Specificity, %	PPV, %	NPV, %	*p* Value
miR-21-5p	0.67	0.35, 0.98	44.44	100.00	100.00	44.44	0.339
miR-24-3p	0.69	0.36, 1.00	77.78	75.00	87.50	60.00	0.289
miR-29a-3p	0.46	0.07, 0.85	57.14	75.00	80.00	50.00	0.865
miR-222-3p	0.58	0.23, 0.94	55.56	75.00	83.33	42.86	0.666
Four miRs panel	0.79	0.48, 1.00	71.43	100.00	100.00	66.67	0.106

**Table 3:** ROC curve analysis characteristics of biomarkers in relation to cardiac comorbidities after radiotherapy (*n* = 10). AUC—area under the curve; CI—confidence interval; PPV—positive predictive value; NPV—negative predictive value.

Panel	AUC	95% CI	Sensitivity, %	Specificity, %	PPV, %	NPV, %	*p* Value
NT-proBNP	0.93	0.79, 1.00	85.71	100.00	100.00	75.00	<0.001
NT-proBNP + miR-21-5p	0.91	0.70, 1.00	85.71	100.00	100.00	75.00	0.004
NT-proBNP + miR-24-3p	0.95	0.82, 1.00	85.71	100.00	100.00	75.00	<0.001
NT-proBNP + miR-29a-3p	1.00	1.00, 1.00	100.00	100.00	100.00	100.00	<0.001
NT-proBNP + miR-222-3p	0.95	0.82, 1.00	85.71	100.00	100.00	75.00	<0.001
NT-proBNP + four miRs panel	1.00	1.00, 1.00	100.00	100.00	100.00	100.00	<0.001

## Data Availability

The datasets used and/or analyzed during the current study are available from the corresponding author on reasonable request.

## Appendix A

**Table A1:** MiRNAs modulated by irradiation (IR) and potentially involved in cardiotoxicity.

No.	miRNA	Experimental Species	Source	Method	Modulation Post-IR (Increased/Decreased)	Type of Disease	Number of Patients/Cell Lines/Rats	Ref.
1	miRNA-1	Rat	Left ventricular tissue	RT-qPCR ^1^	Decreased	-	40	[44]
2		Rat	Left ventricular tissue	RT-qPCR	Decreased	-	12	[59]
3		Rat	Ventricular tissue	RT-qPCR	Decreased in left ventricle but not in the right ventricle	-	10	[29]
4	miRNA-21	Rat	Left ventricular tissue	RT-qPCR	Increased	-	40	[44]
5		Rat	Left ventricular tissue	RT-qPCR	Increased	-	12	[59]
6		Rat	Ventricular tissue	RT-qPCR	Increased in both the left and right ventricles	-	10	[29]
7	miRNA-24	Human	Serum	qPCR profiling	Increased	Breast cancer	28	[60]
8	miRNA-29a	Human	Plasma	Microarray profiling	Decreased	NSCLC ^3^	5	[41]
9		Human	Cell lines	RT-qPCR	Decreased (exosomal expression)	NSCLC	3 (NCI-H460, A549, NCI-H1299)	[41]
10	miRNA-34a	Human	Cell culture	RT-qPCR	Increased	-	1 (human cardiomyocytes)	[42]
11		Mice	Plasma/Tissue	NGS ^2^	Increased	-	192	[61]
12	miRNA-222	Human	Blood	RT-qPCR	Increased	Breast cancer	136	[43]
13		Human	Cell culture	RT-qPCR	Increased at 2 h and reduced at 24 h	-	1 (human umbilical vein endothelial cells)	[62]

^1^ RT-qPCR—reverse transcription quantitative polymerase chain reaction; ^2^ NGS—next-generation sequencing; ^3^ NSCLC—non-small-cell lung cancer.
